# From Phenolic Profile to Gut Function: Comparative Effects of Region-Specific Shilajit on Selected Culturable Intestinal Microbial Groups and β-Glucuronidase Activity—A Preliminary Study

**DOI:** 10.3390/molecules31122172

**Published:** 2026-06-21

**Authors:** Elham Kamgar, Małgorzata Gumienna, Barbara Górna-Szweda, Miroslava Kačániová, Przemysław Łukasz Kowalczewski, Joanna Zembrzuska

**Affiliations:** 1 Department of General and Analytical Chemistry, Institute of Chemistry and Technical Electrochemistry, Faculty of Chemical Technology, Poznan University of Technology, 61-131 Poznań, Poland; elham.kamgar@doctorate.put.poznan.pl; 2 Department of Food Technology of Plant Origin, Faculty of Food Science and Nutrition, Poznań University of Life Sciences, 60-624 Poznań, Poland; malgorzata.gumienna@up.poznan.pl (M.G.); barbara.gorna@up.poznan.pl (B.G.-S.); 3 School of Medical and Health Sciences, VIZJA University, 01-043 Warsaw, Poland; m.kacaniova@vizja.pl; 4 Institute of Horticulture, Faculty of Landscape Engineering, Slovak University of Agriculture in Nitra, 949 76 Nitra, Slovakia; 5 Faculty of Health Sciences, Andrzej Frycz Modrzewski Krakow University, 30-705 Kraków, Poland

**Keywords:** phenolic acids, simulated gastrointestinal digestion, intestinal fermentation, microbial modulation

## Abstract

Shilajit is a complex natural phytomineral substance whose composition and biological activity may vary depending on geographical origin. This study compared three commercially available Shilajit samples from Russia (S1), Nepal (S2), and Iran (S3) in terms of phenolic acid profile, antimicrobial activity, and their effects on selected intestinal microorganisms and β-glucuronidase activity after simulated gastrointestinal digestion. The samples differed markedly in their phenolic composition, with S3 showing the highest total content of the quantified phenolic acids. All samples exhibited antimicrobial activity, although their intensity depended on the microorganism tested. The in vitro digestion model revealed clear sample-dependent effects: S2 showed the lowest net β-glucuronidase activity and the most beneficial modulation of *Lactobacillus* and *Bifidobacterium*, whereas S1 exerted the strongest suppressive effect on *Escherichia coli*. In contrast, S3, despite the richest phenolic profile, showed the highest β-glucuronidase activity. These findings indicate that the biological activity of Shilajit depends not only on the quantified phenolic acids but also on the broader, region-specific chemical matrix of the material.

## 1. Introduction

Shilajit is a Himalayan, resinous phytocomplex exuding from weathered rocks in the Himalayas, Andes, Altai and Caucasus mountains [1,2]. Geologically, it represents the end-product in centuries of humification and is synthesized mainly in the dry mass by humic substances (≈60–80%), specifically low-molecular-weight fulvic acids; it also incorporates humic acid polymers, dibenzo-α-pyrone chromophore pigments (DBPs), and extensive suites of inorganic and organic trace elements such as selenium, zinc and iron complexed in organic ligands [3]. This heterogeneous complex is the reason in traditional Indian and Tibetan Medicine for categorization of Shilajit as a “rasāyana” rejuvenator and gives rationalization for its pleiotropic pharmacology [4,5].

At the molecular level, Shilajit exerts powerful redox-modulating action: fulvic acids and DBPs compete for reactive oxygen and up-regulate the antioxidant axis (Nrf2/HO-1) whilst suppressing concomitantly NF-κB-driven inflammation signalling, effects reproducing in endothelial cells, in macrophages and in neuronal cultures [6,7,8]. DBPs also act as endogenous ubiquinone analogues and, in redox equilibrium, as redox couple with coenzyme Q10; co-administration of purified exudate has been demonstrated in vivo to increase CoQ10 levels in the mitoplasts and accelerate electron-transport kinetics and ATP production, and thus provides rationalization in molecule and in physiology for anti-fatigue and exercise-performance claims so frequently attributed in folklore and advertising to the exudate [9,10,11]. In addition to its redox core, Shilajit raises nitric-oxide bioavailability in the endothelium and lowers malondialdehyde and high-sensitivity C-reactive protein and pulse-wave–derived measurements of vasodilatory stimulus in randomized and controlled human studies, and thus provides support, in addition, for cardiovascular benefit [12,13]. Translation into human physiology of this rationalization in molecules and in biochemistry remains emergent but so far impressive. A double-blind trial in healthy middle-aged men demonstrated that 90 days’ twice-daily purified Shilajit 250 mg increased total and free testosterone and DHEAS in each case significantly but did so in the absence of perturbation of the gonadotropins in accord with traditional ethnomedical use as an aphrodisiac and fertility-enhancing agent [14]. In the neurocognitive domain, standard Andean Shilajit fractions with fulvic acid suppressed tau-protein aggregation in vitro, increased neurite elongation in N2a cells, and suppressed Alzheimer-like disease in preclinical disease models, making the molecule itself a candidate disease-modifying therapy for neurodegeneration. These results, in concert, present Shilajit as a multi-target redox-active adaptogen with experimentally proven action in mitochondrial energetics, endocrine function, blood vessel homeostasis, and neuroprotection [15,16,17].

Undigested or phase-II–conjugated moieties that escape absorption in the small intestine—including residual oligopeptides, simple sugars, short-chain fatty acids (SCFAs), secondary bile acids and glucuronidated xenobiotics or steroid hormones—reach the colon, where they act as both carbon sources and signalling molecules for the resident microbiota. A pivotal functional read-out of this metabolic crossroads is bacterial β-glucuronidase (GUS), an estrobolome-encoded enzyme that hydrolyses glucuronide conjugates, thereby re-liberating aglycone drugs, bilirubin and oestrogens for enterohepatic re-uptake. Clinical studies illustrate its pathophysiological relevance: faecal GUS activity is elevated 1.7- to 12-fold in colorectal cancer patients compared with healthy controls [18], while high GUS levels predict the severity of irinotecan-induced late-onset diarrhoea by deglucuronidating the inactive metabolite SN-38G back to mucosa-toxic SN-38 [18]. Beyond oncology, the enzyme modulates systemic hormone balance; excessive deconjugation of oestrogen glucuronides has been implicated in oestrogen-dominant disorders, positioning GUS as an emerging biomarker and therapeutic target within the estrobolome [19].

Humic and fulvic acids—which constitute >60% of the dry weight of Shilajit [15]—can reshape this enzymatic landscape by altering community composition and metabolic output: oral supplementation with humic acids for 45 days increased total bacterial abundance by ~30% without eroding taxonomic diversity in healthy volunteers [20], and ameliorated dextran-sulphate-induced colitis in mice by restoring barrier integrity and normalising microbial profiles [21]. Although GUS activity has not yet been measured after Shilajit ingestion, the phytocomplex’s high fulvic-acid content, redox capacity, and trace-element profile resemble those of standalone humic preparations, suggesting that Shilajit could similarly shift the microbiome towards a lower-GUS phenotype and thereby enhance xenobiotic detoxification and oestrogen clearance.

In light of the foregoing evidence, and the absence of data on how Shilajit influences the gut ecosystem after simulated gastrointestinal digestion, the present study was designed to (i) characterize the impact of Shilajit samples derived from distinct geographical regions on colonic microbiota composition, (ii) quantify their modulatory effects on bacterial β-glucuronidase (GUS) activity, and (iii) evaluate their antimicrobial potential against representative gut and opportunistic pathogens. In parallel, we verified the total and extractable levels of antioxidant constituents to explore structure-activity relationships underpinning any observed bioeffects.

## 2. Results and Discussion

The three Shilajit samples differed markedly in their PA composition. In all samples, hydroxybenzoic acid derivatives predominated over hydroxycinnamic acid derivatives, with gallic acid being the major compound, followed by vanillic and syringic acids (Table 1). Based on the sum of the quantified compounds, S3 exhibited the richest phenolic profile (approximately 2601 μg/g), followed by S1 (approximately 2248 μg/g), whereas S2 contained the lowest total amount of the analyzed PAs (approximately 1055 μg/g). In addition to its highest gallic and vanillic acid contents, S3 also contained the highest levels of *p*-coumaric, ferulic, syringic, and chlorogenic acids, while S2 lacked detectable ferulic, sinapic, and chlorogenic acids. Thus, the tested Shilajit samples showed pronounced region-dependent differences in the content and distribution of low-molecular-weight PAs. This observation is consistent with recent comparative HPLC-MS/MS data showing substantial geographical variation in the PA composition of Shilajit collected from Iran, India, Nepal, Russia, and Kyrgyzstan, with gallic acid generally being the dominant constituent and hydroxybenzoic acids exceeding hydroxycinnamic acids in most samples [22].

The observed differences are important because they indicate that Shilajit cannot be treated as a chemically uniform material. Rather, it should be considered a region-specific organic-mineral matrix whose composition is shaped by botanical input, environmental conditions, and the degree of humification. This interpretation is also supported by physicochemical studies showing that Shilajit contains a heterogeneous mixture of fulvic and humic substances, mineral constituents, dibenzo-α-pyrones, and phenolic compounds, with marked variability between specimens [3,23]. Therefore, the quantified PAs should be regarded as one relevant chemical fraction of the material rather than as a complete explanation of its biological activity.

All analyzed samples exhibited measurable antimicrobial activity against the tested bacteria and yeasts in the disc diffusion assay, although the magnitude of inhibition depended on both the sample and the microorganism (Table 2). In general, inhibition zones ranged from approximately 10 to 17 mm, indicating broad-spectrum but different activity. S3 showed the strongest inhibition against *Bacillus cereus* and *Staphylococcus aureus*, whereas S2 was the most active against *Candida albicans* and showed slightly stronger inhibition of *Yersinia enterocolitica*. Against *Escherichia coli*, the differences between samples were small, with all three preparations producing relatively similar inhibition zones. These results suggest that the antimicrobial effect of Shilajit is selective rather than uniformly stronger in one sample across all microorganisms.

This pattern supports the view that antimicrobial activity depends on the overall chemical matrix rather than on the concentration of a single PA. Although phenolic acids such as gallic acid are known to exert antibacterial activity against *E. coli* and other pathogens, their effects are strongly influenced by concentration, compound structure, and interactions with the surrounding matrix [24]. In Shilajit, these interactions are likely further modified by humic and fulvic fractions and mineral constituents [25,26]. For this reason, the stronger activity of S3 against selected Gram-positive bacteria should not be interpreted simply as a consequence of its richer phenolic profile, but rather as an emergent property of the whole sample composition [27,28]. Attempting to establish classical structure-activity relationships based solely on the quantified phenolic fraction is therefore insufficient for such a highly complex matrix. The biological and antimicrobial activity of Shilajit must be viewed through the lens of synergism, where humic and fulvic polymers may facilitate the cellular uptake of both phenolic compounds and inorganic trace elements naturally present in the exudate, thereby amplifying or modulating their intrinsic antimicrobial effects.

The three Shilajit samples exerted clearly different effects on β-glucuronidase activity during simulated gastrointestinal digestion (Table 3). At all analyzed stages, S2 showed the lowest values, S1 displayed intermediate activity, and S3 consistently produced the highest β-glucuronidase activity. After the large-intestine stage (18 h, pH 8.0), the values reached 10.964 U/g for S2, 25.141 U/g for S1, and 34.610 U/g for S3, confirming the stability of this ranking throughout the experiment. Because the digestion control values had already been subtracted, these results represent the net effects of the tested Shilajit samples rather than the absolute enzyme activity of the model system.

Notably, these data do not support a simple inverse relationship between total quantified PAs and β-glucuronidase activity. If the measured phenolic fraction were the main driver of this response, the sample richest in PAs would be expected to show the strongest suppressive effect. However, the opposite tendency was observed: S3, which contained the highest total amount of quantified PAs, produced the highest β-glucuronidase activity, whereas S2, which had the lowest phenolic content, showed the lowest enzyme activity. This indicates that the modulation of β-glucuronidase by Shilajit depends on factors beyond the analyzed PAs alone. Furthermore, certain phenolic compounds can serve as fermentable carbon sources for specific gut microbes. Instead of suppressing enzymatic activity, a highly concentrated phenolic pool might inadvertently stimulate the proliferation and metabolic activity of diverse, opportunistic GUS-producing taxa, leading to a net increase in enzyme activity, as observed for S3. The most plausible explanation is that region-specific differences in the broader Shilajit matrix, including humic and fulvic fractions and other non-phenolic constituents, shape the microbial enzymatic response more strongly than the quantified phenolic profile itself [20,21]. At a mechanistic level, humic and fulvic acids—which constitute the predominant fraction of the Shilajit matrix—are rich in functional groups, such as carboxyl and phenolic hydroxyl groups, capable of actively interacting with bacterial cell walls and modulating membrane permeability. These macrostructures can also serve as organic electron acceptors or donors, directly influencing microbial redox balance and metabolic pathways independently of free phenolic monomers. Moreover, the region-specific profile of inorganic trace elements complexed within these organic carriers (e.g., selenium, zinc, and iron) may act as selective enzymatic cofactors or inhibitors, explaining why the S2 matrix significantly down-regulated net β-glucuronidase activity while concurrently promoting a eubiotic enrichment of *Lactobacillus* and *Bifidobacterium* species. From a physiological perspective, this observation is relevant because gut microbial β-glucuronidase is regarded as an important functional marker of microbial metabolism, especially in relation to xenobiotic and estrogen deconjugation [18]. At the same time, the literature shows that β-glucuronidase activity is not attributable to a single bacterial taxon but results from the combined contribution of multiple gut bacteria carrying structurally diverse GUS enzymes [29]. Therefore, differences in enzyme activity may reflect shifts in microbial function rather than merely changes in total bacterial counts.

The microbial counts revealed distinct and functionally different fermentation profiles for the tested samples. S2 promoted the strongest increase in beneficial bacterial groups, especially *Lactobacillus* and *Bifidobacterium*, whose final counts reached 9.380 and 9.374 log CFU/mL, respectively. S3 also stimulated these groups, although slightly less effectively than S2. In contrast, S1 led to the lowest final counts of both *Lactobacillus* and *Bifidobacterium*, but it strongly suppressed *E. coli*, reducing its count from 6.597 to 1.588 log CFU/mL after the large-intestine stage. This effect was not observed for S2 or S3, both of which were associated with a marked increase in *E. coli* during fermentation. Thus, the three samples differed not only in the intensity but also in the direction of their microbiological effects: S1 acted more selectively against *E. coli*, S2 showed the most favorable eubiotic profile, and S3 promoted broader microbial growth without a corresponding functional advantage in terms of β-glucuronidase activity.

The S2 profile appears the most beneficial from a gut-function perspective because it combines the lowest β-glucuronidase activity with the highest final counts of *Lactobacillus* and *Bifidobacterium*. Such a pattern is generally consistent with the broader literature on polyphenols and humic substances, both of which can promote beneficial bacterial groups and improve intestinal homeostasis. Experimental studies on humic acids have shown positive modulation of gut microbiota, including increases in *Lactobacillus* and *Bifidobacterium* in inflammatory models [20,21], while polyphenol-rich matrices are commonly associated with enrichment of beneficial bacteria and suppression of potentially harmful taxa [30,31]. Nevertheless, the present data also show that this favorable effect cannot be explained by a simple “more phenolics = better microbiological outcome” model, since S2 had the poorest quantified phenolic profile.

The relationship between *E. coli* abundance and β-glucuronidase activity was only partial. The strong suppression of *E. coli* by S1 was accompanied by lower β-glucuronidase activity than in S3, which is biologically plausible because *E. coli* typically carries the uidA gene encoding β-glucuronidase [32]. However, S2 still exhibited the lowest β-glucuronidase activity despite the increase in *E. coli* counts, which indicates that *E. coli* alone did not determine the enzymatic outcome in this model. This finding agrees with reports showing that β-glucuronidase is widely distributed among gut microorganisms and that its functional expression depends on the abundance and diversity of GUS-producing taxa rather than on the presence of a single species [19,29].

The data indicate that the biological effects of Shilajit after simulated digestion were strongly sample-dependent and only weakly predictable from the quantified PA profile (Table 4). S3 was chemically the richest in the analyzed PAs, yet it showed the highest β-glucuronidase activity and did not provide the most favorable microbiological profile. In contrast, S2, despite its lowest PA content, produced the lowest β-glucuronidase activity together with the strongest stimulation of *Lactobacillus* and *Bifidobacterium*. S1 showed a different mode of action, characterized mainly by a marked suppression of *E. coli*. Therefore, the studied Shilajit samples appear to exert distinct functional effects that reflect differences in the entire chemical matrix rather than in PAs alone. This conclusion is scientifically important because it argues against overinterpreting phenolic composition as the sole determinant of post-digestion bioactivity. While PAs such as gallic, caffeic, ferulic, *p*-coumaric, and chlorogenic acids are biologically relevant and may contribute to microbiota modulation, they are themselves subject to microbial biotransformation, and their effects are highly context-dependent [33]. For example, chlorogenic acid can be hydrolyzed by selected gut bacteria, including bifidobacteria, whereas hydroxycinnamic acids and other polyphenols may alter the intestinal ecosystem through both direct antimicrobial action and indirect effects on barrier function and inflammation [34,35]. In a complex material such as Shilajit, these mechanisms likely operate alongside the activity of humic and fulvic components, producing sample-specific functional outcomes [20]. Among the tested samples, S2 demonstrated the most advantageous profile in the context of gut function, because it combined stimulation of beneficial bacterial groups with the strongest reduction in net β-glucuronidase activity. S1 may also be of interest due to its pronounced anti-*E. coli* effect. In contrast, S3, despite the richest PA composition, appeared less favorable in functional terms. These findings support the need for deeper chemical standardization of Shilajit and suggest that future studies should focus not only on PAs but also on humic/fulvic fractions and other region-specific constituents responsible for gut-related biological effects.

## 3. Materials and Methods

### 3.1. Research Materials

This study exploited three raw, commercially available Shilajit samples, designated S1 to S3, with different geographical origins: S1—Russia, S2—Nepal, and S3—Iran. These specific regions were selected because they represent distinct, major geographical and geological mountainous systems (Altai, Himalayas, and the Zagros/Caucasus network, respectively) historically renowned for Shilajit exudation. Comparing these origins allows for a comprehensive evaluation of how diverse environmental factors and botanical inputs shape the organic-mineral matrix and its subsequent biological effects. Furthermore, these samples reflect the primary authentic origins currently available on the commercial market.

### 3.2. Antioxidants Extraction Procedure

Approximately 1 g of freeze-dried sample was accurately weighed and combined with 9 mL of 80% methanol (*v*/*v*). The suspension was vigorously shaken for 20 min to facilitate the extraction of soluble compounds. Following this, the mixture was centrifuged at 10,000× *g* for 15 min at 4 °C. The clear supernatant was collected and passed through a 0.22 μm PTFE syringe filter (Alchem, Toruń, Poland) to remove particulate matter before analysis.

### 3.3. Phenolic Acid Determination

The concentration of selected phenolic acids (PAs)—including *p*-coumaric, vanillic, gallic, caffeic, ferulic, syringic, sinapic, and chlorogenic acids—in Shilajit was quantified following a protocol adapted from Kamgar et al. [22]. The analysis was performed using an UltiMate 3000 RSLC system (Dionex™, Thermo Scientific Inc., Waltham, MA, USA) coupled to an API 4000 QTRAP triple-quadrupole mass spectrometer (AB Sciex, Foster City, CA, USA) equipped with an electrospray ionization (ESI) source operating in positive ion mode (UHPLC-MS/MS, Dionex™, Thermo Scientific Inc., Waltham, MA, USA). Chromatographic separation was achieved on a Luna C18 column (3 µm, 150 mm × 2.0 mm I.D., Phenomenex Inc., Torrance, CA, USA). The column temperature was maintained at 35 °C, and the injection volume was 5.0 μL. The mobile phase consisted of 5 mM ammonium acetate in water (solvent A) and methanol (solvent B). The gradient program started at 90% B (0.0–2.5 min), increased to 100% B at 3.5 min, and was held for 0.5 min. The flow rate was 0.20 mL/min. A post-run equilibration time of 4.0 min was applied before the next injection.

The mass spectrometry operating conditions for all PAs were as follows: curtain gas 10 psi; nebulizer gas and auxiliary gas 40 psi; source temperature 400 °C; ion spray voltage −4500 V; and collision gas set to medium. Quantitative analysis was conducted in multiple reaction monitoring (MRM) mode. For each analyte, one transition of the deprotonated molecular ion and its corresponding product ion was monitored. The second transition confirmed the identification of each acid. These transitions (*m*/*z*), along with the associated declustering potential (DP), collision energy (CE), and collision cell exit potential (CXP), are summarized in Table 5.

PAs in the methanolic extracts of Shilajit were quantified using a four-point standard addition method. The spiking concentrations were adjusted according to the expected levels of each phenolic acid in the samples. Quantification was based on the peak areas of the respective analytes.

### 3.4. Antimicrobial Activity of Shilajit

The antimicrobial activity of the Shilajit samples was evaluated using the disc diffusion method by measuring the zones of growth inhibition of the selected microorganisms. Appropriate culture media, specifically Mueller–Hinton agar (MHA, Oxoid, Basingstoke, UK) for bacterial strains and Sabouraud’s dextrose agar (SDA, Oxoid, Basingstoke, UK) for yeast strains, were prepared in Petri dishes. Before the assay, the microorganisms were precultured; bacterial strains were incubated in Mueller–Hinton broth (MHB, Oxoid, Basingstoke, UK) at 37 °C for 24 h, whereas yeast strains were incubated in Sabouraud’s dextrose broth (SDB, Oxoid, Basingstoke, UK) at 25 °C for 24 h. The microbial suspensions were subsequently adjusted to a turbidity of 0.5 McFarland standard, corresponding to approximately 1.5 × 10^8^ colony-forming units per milliliter (CFU/mL).

An aliquot of 100 μL of the standardized microbial suspension was uniformly spread onto the surface of the respective agar plates. Sterile blank paper discs (6 mm in diameter; Oxoid, Basingstoke, UK) were placed on the inoculated plates, and 10 μL of the Shilajit extract was applied to each disc. The plates were then incubated for 24 h at 37 °C for bacteria and 25 °C for yeasts. Following incubation, the radii of the growth inhibition zones induced by the Shilajit samples were measured in millimeters. All assays were performed in triplicate, and the results were expressed as the mean value ± standard deviation (SD).

Both positive and negative controls were included in the experimental design. Positive controls consisted of standard antimicrobial discs: cefoxitin for Gram-positive bacteria, gentamicin for Gram-negative bacteria, and fluconazole for yeasts (all sourced from Oxoid, Basingstoke, UK). Sterile blank discs without the Shilajit extract served as the negative control. Based on the measured zones of inhibition, the antimicrobial efficacy of the Shilajit samples was classified as weak (0–5 mm), moderate (5–8 mm), or strong (>8 mm).

### 3.5. In Vitro Digestion Process

The in vitro digestion of Shilajit was carried out using a glass bioreactor equipped with four ports to allow pH monitoring with an electrode, active pH regulation, precise dosing of biochemical agents and media, and sampling for analytical purposes. The digestion mixture was prepared by dissolving 11.5 g of Shilajit in demineralized water to a final volume of 230 mL. The bioreactor maintained a constant temperature of 37 °C. The experimental design simulated the sequential stages of the gastrointestinal tract: stomach, small intestine, and large intestine. The digestion parameters were established based on previous studies [30,31]. Stomach phase: digestion began by mimicking gastric conditions. A solution containing 60,000 U of pepsin (Sigma-Aldrich, St. Louis, MO, USA) in 2 mL of 0.1 M HCl was added to the sample. The pH was adjusted to 2.0, and digestion was allowed to proceed for 2 h. Small intestine phase: Following the gastric phase, the pH was raised to 6.0 using 1 M NaHCO_3_. Next, 10 mL of a mixture containing 0.02 g of pancreatic extract and 0.12 g of bile salts (both from Sigma-Aldrich), dissolved in 10 mL of 0.1 M NaHCO_3_, was added. The pH was further adjusted to 7.4 with 1 M NaHCO_3_, and human intestinal microbiota, prepared according to the method previously described by Gumienna et al. [36], was introduced at a concentration of approximately 10^6^ CFU/mL. Briefly, the human intestinal microbiota was derived from fresh fecal samples obtained from healthy, adult volunteers who had not received any antibiotic treatment in the six months prior to the study. The samples were pooled, homogenized in an anaerobic phosphate-buffered saline solution, and immediately utilized to ensure high microbial viability. This phase lasted for 2.5 h. Large intestine phase: Finally, the pH was adjusted to 8.0 by adding 1.25 M NaHCO_3_, and fermentation was continued for another 18 h.

### 3.6. Determination of β-Glucuronidase Activity

The β-glucuronidase activity was determined using a modified procedure based on the methods described by Djouzi and Andrieux [37] and Kapnoor and Mulimani [38]. A 200 µL aliquot of the digested Shilajit sample was collected from the bioreactor and mixed with 1500 µL of phosphate buffer (pH 7.0) containing 0.1 M NaCl. The mixture was shaken for 1 h and subsequently centrifuged at 3000 rpm for 15 min. The resulting supernatant (200 µL) was combined with 200 µL of *p*-nitrophenyl-β-D-glucuronide solution (1 mg/mL, prepared in phosphate buffer at pH 6.7). The reaction mixture was incubated at 40 °C for 2.5 h. To terminate the reaction, 2000 µL of sodium carbonate solution was added. The absorbance was measured at 420 nm. The enzymatic activity of β-glucuronidase was expressed as micromoles of *p*-nitrophenol produced per minute per gram of digested material (U/g digested material).

### 3.7. Effect on Intestinal Microflora

To assess the impact of gastrointestinal conditions on microbial growth during the digestion of Shilajit, microbial counts were evaluated at two key time points: after 2 h of incubation (representing small-intestinal conditions, pH 7.4) and after the digestion process (the next 18 h). The experimental model was inoculated with gut microbiota obtained according to the methodology described in Section 3.5. The analysis focused on specific microbial groups: Enterobacteriaceae (cultured on MacConkey selective medium; Sigma Aldrich, Saint Louis, MO, USA), *Lactobacillus* spp. (MRS agar; Sigma Aldrich), *Enterococcus* spp. (agar medium containing kanamycin, esculin, and sodium azide), and *Bifidobacterium* spp. (Garche medium; Sigma Aldrich). The inoculated media were incubated under anaerobic conditions appropriate for each microbial group at 37 °C for 48 to 72 h. The viable counts of microorganisms were determined using the plate count method according to Koch. Viable counts were determined by the standard plate count method and expressed as log CFU/mL of digesta. All analyses were performed in 3 independent experiments with 3 technical replicates.

### 3.8. Statistical Analysis

Statistical analysis of the data was performed using Statistica 13 (Dell Software Inc., Round Rock, TX, USA). For every test, three independent measurements were taken, unless stated otherwise. Prior to conducting the ANOVA, the fundamental statistical assumptions were verified: the normality of data distribution was tested using the Shapiro–Wilk test, and the homogeneity of variances was assessed using Levene’s test. All measurements were analyzed using one-way analysis of variance, with a separate analysis for each dependent variable. Post hoc Tukey honest significant difference (HSD) multiple comparison tests were used to identify statistically homogeneous subsets at α = 0.05.

## 4. Conclusions

Shilajit samples of different geographical origins differed markedly in their PA composition and in their effects on intestinal microbial activity after simulated digestion. Notably, the sample with the richest quantified phenolic profile (S3) did not show the most favorable gut-related response, whereas S2, despite the lowest total content of the analyzed PAs, exhibited the lowest net β-glucuronidase activity and the most beneficial modulation of selected intestinal microorganisms, particularly *Lactobacillus* and *Bifidobacterium*. In turn, S1 showed the strongest suppressive effect on *Escherichia coli*. Overall, these findings indicate that the biological activity of Shilajit cannot be explained solely by the abundance of the quantified PAs, but rather depends on the broader, region-specific chemical matrix of the material. Therefore, careful source selection and deeper chemical standardization of Shilajit are essential for its potential nutraceutical and functional applications. However, it is important to acknowledge that these findings are based on an in vitro digestion model. While this approach provides valuable mechanistic insights into gut microbiome modulation, subsequent in vivo studies and human clinical trials are essential to fully substantiate the systemic health benefits and confirm the clinical efficacy of Shilajit as a nutraceutical.

## Figures and Tables

**Table 1:** Results of the antioxidants composition of the analyzed Shilajit (ug/g).

Acid	S1	S2	S3
*p*-coumaric	55.85 ± 2.29 ^c^	67.94 ± 3.94 ^b^	101.02 ± 1.06 ^a^
Vanillic	549.64 ± 5.55 ^b^	178.84 ± 7.22 ^c^	712.08 ± 6.88 ^a^
Gallic	1191.57 ± 12.80 ^b^	611.51 ± 21.52 ^c^	1405.62 ± 83.80 ^a^
Caffeic	239.77 ± 5.53 ^a^	121.10 ± 2.38 ^b^	135.59 ± 7.41 ^b^
Ferulic	25.23 ± 3.36 ^b^	nd	59.15 ± 2.96 ^a^
Syringic	146.21 ± 2.80 ^b^	75.52 ± 2.37 ^c^	160.50 ± 5.46 ^a^
Sinapic	22.11 ± 2.38	nd	nd
Chlorogenic	17.67 ± 0.46 ^b^	nd	27.10 ± 3.37 ^a^

Mean values with the same letters in the row were not significantly different (α = 0.05). nd—not detected.

**Table 2:** The results of antimicrobial activity (mm).

Sample	*Bacillus cereus*	*Vibrio parahemoliticus*	*Listeria monocytogenes*	*Yersinia enterocolitica*	*Staphylococcus aureus*	*Eschcerichia coli*	*Candida albicans*	*Candida krusei*	*Candida glabrata*	*Candida tropicalis*
S1	15.67 ± 0.58	15.33 ± 0.58	14.67 ± 0.37	11.33 ± 0.58	15.67 ± 0.58	12.33 ± 0.37	13.00 ± 1.00	11.67 ± 0.58	10.67 ± 0.58	12.33 ± 1.15
S2	13.33 ± 0.37	15.33 ± 1.15	14.33 ± 0.58	12.33 ± 0.37	16.33 ± 1.73	12.00 ± 1.00	15.00 ± 1.73	10.67 ± 1.15	10.67 ± 0.58	12.67 ± 0.37
S3	17.33 ± 0.59	12.00 ± 1.00	14.33 ± 0.58	11.00 ± 1.00	17.33 ± 0.58	12.67 ± 1.15	12.67 ± 0.58	10.67 ± 0.58	10.33 ± 0.37	12.33 ± 1.15

**Table 3:** The results of β-Glucuronidase activity (U/g).

The Stages of Digestion	S1	S2	S3
2 h at pH 7.4 “after small intestine”	22.030 ± 0.751 ^b^	8.638 ± 0.032 ^c^	30.992 ± 0.411 ^a^
pH 8.0 “in the large intestine”	21.933 ± 1.259 ^b^	9.384 ± 0.040 ^c^	30.957 ± 0.296 ^a^
18 h at pH 8.0 “after the large intestine”	25.141 ± 0.999 ^b^	10.964 ± 0.075 ^c^	34.610 ± 0.292 ^a^

Mean values with the same letters in the row were not significantly different (α = 0.05).

**Table 4:** Quantitative changes in the intestinal microflora during digestion of the analyzed Shilajit [log 10 CFU/mL].

Microorganism	S1			S2			S3		
	pH 7.4 ^1^	2 h pH 7.4 ^2^	18 h pH 8.0 ^3^	pH 7.4 ^1^	2 h pH 7.4 ^2^	18 h pH 8.0 ^3^	pH 7.4 ^1^	2 h pH 7.4 ^2^	18 h pH 8.0 ^3^
*Lactobacillus*	7.752 ± 0.102	7.4430 ± 0.075	8.021 ± 0.081	7.276 ± 0.093	7.437 ± 0.068	9.380 ± 0.507	8.269 ± 0.293	8.477 ± 0.323	9.203 ± 0.408
*Enterococcus*	6.698 ± 0.066	6.423 ± 0.101	7.874 ± 0.069	6.998 ± 0.121	7.455 ± 0.071	7.404 ± 0.209	8.188 ± 0.137	7.349 ± 0.137	6.385 ± 0.091
*E. coli*	6.597 ± 0.090	5.351 ± 0.058	1.588 ± 0.047	5.929 ± 0.119	6.078 ± 0.099	9.505 ± 0.449	5.929 ± 0.091	7.439 ± 0.304	9.567 ± 0.192
*Bifidobacterium*	8.167 ± 0.075	8.366 ± 0.609	7.952 ± 0.207	7.216 ± 0.382	7.439 ± 0.304	9.374 ± 0.451	8.380 ± 0.142	8.562 ± 0.397	9.145 ± 0.408

^1^ “in small intestine” with fecal flora; ^2^ “after small intestine”; ^3^ “after the large intestine”.

**Table 5:** Optimal operating parameters of the tandem mass spectrometer for the tested compounds.

Compound	[M-H]^−^	DP (V)	MRM 1/MRM2	CE (V)	CXP (V)
Caffeic acid	179	−51	179 → 135 179 → 106.3	−22/−32	−7/−7
Chlorogenic acid	353	−65	353 → 191 353 → 85	−24/−64	−9/−5
Ferulic acid	193	−55	193 → 134 193 → 178	−20/−18	−7/−9
Gallic acid	169.1	−55	169 → 125 169 → 79	−22/−32	−9/−5
Vanillic acid	167	−50	167 → 107.9 167 → 122.9	−26/−18	−7/−21
*p*-coumaric acid	163	−55	163 → 119 163 → 92.9	−24/−44	−9/−3
Syringic acid	197	−65	197 → 120.9 197 → 153	−24/−18	−9/−9
Sinapic acid	223	−60	223 → 148.7 223 → 163.8	−28/−20	−25/−7

## Data Availability

Data will be made available on request.
